# Efficacy and Cardiovascular Adverse Effects of Erythropoiesis Stimulating Agents in the Treatment of Cancer-Related Anemia: A Systematic Review of Randomized Controlled Trials

**DOI:** 10.7759/cureus.17835

**Published:** 2021-09-08

**Authors:** Sanjay Rao Gergal Gopalkrishna Rao, Seif Bugazia, Tamil Poonkuil Mozhi Dhandapani, Anjli Tara, Ishan Garg, Jaimin N Patel, Jimin Yeon, Marrium S Memon, Abilash Muralidharan, Safeera Khan

**Affiliations:** 1 Internal Medicine, California Institute of Behavioral Neurosciences & Psychology, Fairfield, USA; 2 Internal Medicine, Kasturba Medical College, Manipal, IND; 3 Faculty of Medicine, University of Benghazi, Benghazi, LBY; 4 Internal Medicine/Family Medicine, California Institute of Behavioral Neuroscience & Psychology, Fairfield, USA; 5 Medicine, Kanyakumari Government Medical College, Nagercoil, IND; 6 General Surgery, California Institute of Behavioral Neurosciences & Psychology, Fairfield, USA; 7 General Surgery, Liaquat University of Medical and Health Sciences, Jamshoro, PAK; 8 Medicine, California Institute of Behavioral Neurosciences & Psychology, Fairfield, USA; 9 Department of Medicine, Ross University School of Medicine, Miami, USA; 10 Medicine, St. Martinus University Faculty of Medicine, Curacoa, CUW; 11 College of Medicine, Hanyang University, Seoul, KOR; 12 Research, California Institute of Behavioral Neurosciences & Psychology, Fairfield, USA; 13 Internal Medicine, State University of New York (SUNY) Downstate Health Science Center, New York, USA

**Keywords:** erythropoiesis stimulating agents, anemia, cancer, epoetin, darbepoetin, hemoglobin, blood transfusion, thromboembolism, cardiovascular side effects

## Abstract

Anemia is a common complication of cancer. Treatment of anemia in cancer is crucial as anemia adversely affects the quality of life, therapeutic outcomes, and overall survival. Erythropoiesis stimulating agents (ESAs) are valuable drugs for treating cancer-related anemia. Cardiovascular adverse effects are a significant concern with ESA therapy, and there is wide variability in therapeutic goals and characteristics of patients who undergo treatment with ESAs. As a result, a careful analysis of the currently available data on the efficacy and safety of these drugs is necessary. This data analysis will aid in the rational use of ESAs for the treatment of anemia in cancer. The objective of this systematic review is to elucidate the pathogenesis of anemia in cancer, assess the effectiveness of ESAs in treating anemia in cancer, and the overall risk of cardiovascular adverse effects associated with the use of ESAs and their impact on prognosis.

We searched literature from online databases - PubMed, PubMed Central, MEDLINE, Cochrane Library, and clinical trials register (clinicaltrials.gov) to identify prospective phase II and phase III randomized controlled trials (RCTs). We chose RCTs that directly compared patients with cancer who were treated with ESAs to those who were not treated with ESAs. January 2008 was taken as the lower date limit and May 2021 as the upper date limit. Only English language literature and human studies were included. The quality appraisal was completed using the Cochrane risk bias assessment tool, and data from a total of 10,738 patients with cancer in 17 RCTs were identified and included for systematic review.

Our review concludes that ESAs effectively reduce the necessity for blood transfusions and increase mean hemoglobin levels in anemic cancer patients. ESA therapy is associated with cardiovascular adverse effects, including venous thromboembolism, thrombophlebitis, hypertension, ischemic heart disease, cardiac failure, arrhythmia, arterial thromboembolism, and cardiac arrest. Aggressive ESA dosing to achieve higher hemoglobin levels and preexisting uncontrolled hypertension increases these cardiovascular side effects. Venous thromboembolism is the most significant adverse effect attributed to ESA therapy. However, there is no major change in overall survival with ESA therapy, and administration of ESAs can be carried out in anemic cancer patients with careful assessment of thromboembolism risk factors, risk-benefit ratio, and monitoring of hemoglobin levels.

## Introduction and background

Anemia is a common complication among cancer patients. It adversely affects the quality of life and is a strong predictor of reduced survival in cancer patients [1]. The pathogenesis of anemia in cancer is multifactorial. Anemia can develop due to the underlying malignant disease, comorbidities such as nutritional deficiencies, blood loss, renal insufficiency, or as a consequence of myelosuppressive chemotherapy, radiotherapy, or a combination of all of these [2]. The type of cancer, the stage and duration of disease, the intensity and type of tumor therapy, and the occurrence of intercurrent infections or surgery all influence anemia in cancer [3].

Anemia affects patients with cancer in several ways. Fatigue is the most debilitating symptom. Other symptoms include impaired cognitive function, exertional dyspnea, nausea, anorexia, syncope, confusion, and depression [4]. The degree of anemia, the rapidity of its onset, and the patient's pulmonary and cardiovascular function determine the severity of the symptoms [5]. Treatment is based on the cause and the degree of anemia. The goal of therapy is to increase hemoglobin levels and correct the underlying cause [6]. The correction of anemia in cancer patients improves the quality of life, increases treatment efficacy, and has the potential to improve survival [7,8]. 

Erythropoiesis stimulating agents (ESAs), epoetin, and darbepoetin are widely used to treat anemia in cancer. Treatment of anemia in cancer patients using ESAs significantly improves the quality of life, predominantly by reducing fatigue [9]. Although ESAs are valuable drugs for treating anemia in cancer patients, they have been reported to increase the risk of thromboembolism [10,11]. However, data on other cardiovascular adverse effects with the use of ESAs is limited. Cardiovascular adverse effects are a major concern with ESA therapy in treating anemia in cancer to impact survival. Also, therapeutic aims and characteristics of patients treated with ESAs vary greatly, necessitating a careful analysis of the currently available data on the efficacy and safety of these drugs. This data analysis helps in our ability to rationally use ESAs for the treatment of anemia in cancer.

This systematic review elucidates the pathogenesis of anemia in cancer, the effectiveness of ESAs in treating anemia in cancer, and the overall risk of cardiovascular adverse effects associated with the use of ESAs and their impact on prognosis. 

## Review

Methods

Protocol

This systematic review is in accordance with the Preferred Reporting Items for Systematic Reviews and Meta-Analyses (PRISMA) 2020 guidelines [12].

Publication Search

A methodical search of the electronic databases PubMed, PubMed Central, and MEDLINE was conducted on May 10, 2021. Keywords included in our search were "Recombinant erythropoietin," "Epoetin alfa'', "Darbepoetin alfa'', "Cancer," and "Anemia." For each keyword, the relevant terms of the medical subject heading (MeSH) were identified. Each keyword was combined with its MeSH terms using the Boolean operator "OR" and searched in the database. Different keywords and their relevant MeSH terms were combined using the Boolean operator "AND" to get relevant studies. A total of 1635 studies were identified. We also searched Cochrane Library using the keywords and identified 66 relevant studies. Eleven relevant studies identified from the clinical trials register (clinicaltrials.gov) were also considered for review. The inclusion/exclusion criteria were applied, and randomized controlled trials (RCTs) from January 2008 to May 2021 were identified. The search was extended to studies published in the English language literature for human participants. 

The Final Search Strategy

Recombinant erythropoietin OR Epoetin alfa OR Darbepoetin alfa OR ("Epoetin Alfa/administration and dosage"[Mesh] OR "Epoetin Alfa/adverse effects"[Mesh] OR "Epoetin Alfa/analogs and derivatives"[Mesh] OR "Epoetin Alfa/therapeutic use"[Mesh]) OR ("Darbepoetin alfa/administration and dosage"[Mesh] OR "Darbepoetin alfa/adverse effects"[Mesh] OR "Darbepoetin alfa/therapeutic use"[Mesh]) AND Cancer OR Malignancy OR Neoplasms OR ("Neoplasms/abnormalities"[Mesh] OR "Neoplasms/blood"[Mesh] OR "Neoplasms/complications"[Mesh] OR "Neoplasms/drug effects"[Mesh] OR "Neoplasms/drug therapy"[Mesh]) AND Anemia OR low hemoglobin OR ("Anemia/drug effects"[Mesh] OR "Anemia/drug therapy"[Mesh] OR "Anemia/therapy"[Mesh]) combinations were used.

Inclusion/Exclusion Criteria

The literature search was done to identify randomized controlled trials that directly compared patients with cancer treated with and without erythropoiesis stimulating agents (ESAs). Only English language literature and human studies were included. We chose January 2008 as the lower date limit and May 2021 as the upper date limit. Clinical trials that met the following requirements were included: Cancer patients enrolled in prospective phase II and phase III randomized controlled trials; In addition to concomitant chemotherapy and /or radiotherapy, participants with random assignment to ESA treatment or control/placebo; Available data including event or incidence of cardiovascular adverse effects and sample size for analysis. 

Following clinical trials were excluded: Phase I and single-arm phase II randomized controlled trials due to their lack of control groups; Trials with marked inequality of characteristics between groups at baseline; Trials without available data on cardiovascular adverse events. 

Data Extraction

All titles, abstracts, and full-text articles were screened by two reviewers independently. The items extracted from each study included study-year, type of recombinant erythropoietin, number of patients in treatment and control/placebo group, duration of treatment, concomitant treatments, and cancer types. Data on hemoglobin response and blood transfusion requirements were extracted from the results section of each trial. Incidences of cardiovascular adverse effects were extracted from the safety profile in each trial. Another reviewer scrutinized the data extracted by the first reviewer for accuracy. Any disagreements among the reviewers were settled by consensus.

Results

Search Outcome

**Figure 1:**
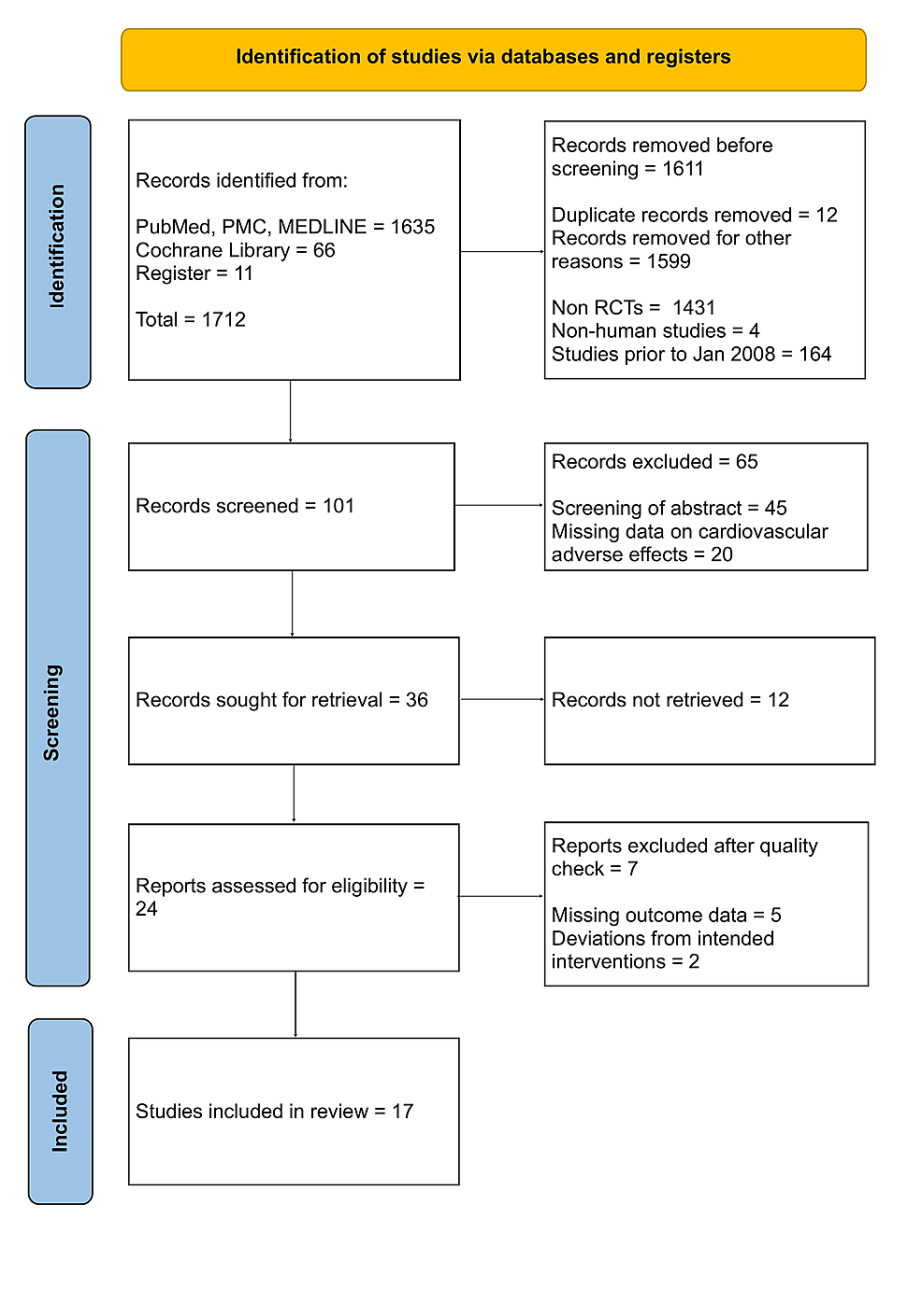
PRISMA Flow Diagram PRISMA = Preferred Reporting Items for Systematic Reviews and Meta-Analyses; PMC = PubMed Central; RCTs = Randomized Controlled Trials

A total of 1701 records were identified through database searches, and 11 additional records were identified through the clinical trials register (clinicaltrials.gov). Out of 1712 records, 12 duplicates were removed. After applying inclusion/exclusion criteria, a total of 1599 records were excluded from 1700 records, including case reports, review articles, phase I studies, single-arm phase II studies, meta-analyses, observational studies, studies published before January 2008, and non-human studies. A total of 101 studies were screened. Out of 101 studies, 65 studies were excluded with reasons after careful screening of abstract, results, and safety profile. As full-text articles were unavailable, additional 12 studies were excluded, and 24 relevant studies were assessed for quality appraisal. The Cochrane Risk Bias Assessment tool was used to assess the quality appraisal of 24 studies. A total of 17 studies were selected for inclusion in the review after the quality assessment. Figure 1 demonstrates the PRISMA flow diagram and the steps taken in screening and selection of studies. 

Characteristics of the 17 randomized controlled trials included in the review are portrayed in Table 1.

**Table 1:** Characteristics of randomized controlled trials included in the review ESA = Erythropoiesis stimulating agent; ALL = Acute lymphocytic leukemia; NSCLC = Non-small cell lung cancer; SCLC = Small cell lung cancer

Year of study	Drug	Patients sample ESA/Control	Treatment Duration	Concomitant treatment	Types of cancer
Aapro - 2008 [13]	Epoetin beta	463 (231/231)	24 weeks	Chemotherapy	Metastatic breast cancer
Blohmer - 2011 [14]	Epoetin alfa	264 (128/129)	NA	Chemoradiotherapy	Cervical cancer
Cabanillas - 2012 [15]	Epoetin alfa	109 (55/54)	NA	Chemotherapy	ALL, lymphoblastic leukemia, Burkitt lymphoma
Engert - 2010 [16]	Epoetin alfa	1303 (648/655)	NA	Chemotherapy	Advanced stage Hodkin's lymphoma
Fujisaka - 2011 [17]	Epoetin beta	181 (89/92)	12 weeks	Chemotherapy	Lung or gynecological cancer
Gascon - 2019 [18]	Darbepoetin alfa	2516 (1680/836)	NA	Chemotherapy	Stage IV NSCLC
Gupta - 2009 [19]	Epoetin beta	115 (58/57)	Five weeks	Chemoradiotherapy	Advanced cervical cancer
Hernandez - 2009 [20]	Darbepoetin alfa	386 (194/192)	16 weeks	Chemotherapy	Lung, ovarian, cervical, breast, and others
Hoskin - 2009 [21]	Epoetin alfa	300 (151/149)	NA	Radiotherapy	Head and neck cancer
Leyland - Jones - 2016 [22]	Epoetin alfa	2098 (1050/1048)	NA	Chemotherapy	Metastatic breast cancer
Pirker - 2008 [23]	Darbepoetin alfa	600 (301/299)	Nine weeks	Chemotherapy	Extensive stage SCLC
Platzbecker - 2017 [24]	Darbepoetin alfa	146 (97/49)	24 weeks	Chemotherapy	Myelodysplastic syndromes
Pronzato - 2010 [25]	Epoetin alfa	216 (107/109)	Four weeks	Chemotherapy	Breast cancer
Ray Coquard - 2009 [26]	Epoetin alfa	218 (110/108)	12 weeks	Chemotherapy	Non-Hodgkin's lymphoma
Smith Jr - 2008 [27]	Darbepoetin alfa	989 (517/472)	16 weeks	No chemotherapy or radiotherapy	Non-myeloid malignancy
Tsuboi - 2009 [28]	Epoetin beta	120 (62/58)	Eight weeks	Chemotherapy	Lung cancer or malignant lymphoma
Untch - 2011 [29]	Darbepoetin alfa	714 (396/318)	NA	Chemotherapy	Primary breast cancer
Total		10,738			

Epoetin alfa or epoetin beta was evaluated in 11 trials with 5,387 patients and darbepoetin alfa in six trials with 5,351 patients. Concomitant treatment varied between trials as follows: chemotherapy (13 trials), radiotherapy (one trial), chemoradiotherapy (two trials), and no treatment (one trial). Eleven trials included 8,807 patients with a single cancer diagnosis [lung cancer (two trials), breast cancer (four trials), head and neck cancer (one trial), cervical cancer (two trials), and non-Hodgkin's lymphoma (two trials)].

Discussion

Pathogenesis of Anemia in Cancer

The pathogenesis of anemia in cancer is multifactorial. Anemia can be caused by chronic inflammation and the synthesis of pro-inflammatory cytokines by both immune and cancer cells [30]. Chronic inflammation leads to suppression of erythropoiesis in bone marrow, shortened erythrocyte survival, increased erythrocyte destruction, changes in iron metabolism due to an increase in hepcidin, and iron-restricted erythropoiesis [31]. Pro-inflammatory cytokines in cancer cause macrophage activation and increased destruction of erythrocytes. Iron restriction and the direct inhibitory action of cytokines on erythropoietic progenitors leads to suppression of erythropoiesis. A direct effect of pro-inflammatory cytokines [interleukin (IL)-1, IL-6, tumor necrosis factor (TNF)-alpha] on kidney cells that produce erythropoietin can cause impairment of erythropoietin synthesis [32], and erythropoietin levels are inadequately low for hemoglobin levels in cancer [33]. The cytokine IL-6 induces hepatic synthesis of hepcidin, which regulates iron homeostasis by controlling the degradation of the iron export protein ferroportin-1, which prevents iron absorption from the small intestine and release of iron from macrophages. Iron is therefore unavailable for erythropoiesis [34]. In addition to cytokine production, cancer itself can cause anemia by suppressing hematopoiesis through bone marrow infiltration. Poor nutritional status is another factor that contributes to anemia in cancer. Patients with advanced cancer often have nausea, vomiting, loss of appetite, and deficiencies of iron, vitamin B12, and folate essential for erythropoiesis. Blood loss associated with cancer surgery or bleeding within the tumor causes anemia [35]. Also, antineoplastic therapies (chemotherapy and radiotherapy) can lead to anemia. Radiation therapy leads to anemia by causing damage to the bone marrow. Chemotherapeutic agents induce anemia by impairing hematopoiesis. Some chemotherapeutic agents are toxic to bone marrow, while others are nephrotoxic, which leads to reduced erythropoietin production by the kidney [36]. 

Effectiveness of ESA Therapy: Reduction in Blood Transfusion

Acute or chronic anemia is often associated with the development of tumor hypoxia. In this condition, tumor cells in the hypoxic areas of solid tumors are resistant to conventional chemotherapy and radiotherapy [37]. Tumour hypoxia adversely impacts radiotherapy or combined radiotherapy-chemotherapy outcomes and chronic hypoxia, caused by anemia, decreases the efficacy of chemotherapy and radiotherapy [38]. Therefore, prevention and treatment of anemia in cancer is a major goal in managing cancer patients. 

Anemia in cancer is primarily treated with packed red blood cell (PRBC) transfusion or administration of erythropoiesis-stimulating agents (ESAs). Hemoglobin and hematocrit levels increase quickly after a packed red blood cell transfusion. However, transfusion carries the risk of transfusion-related reactions, congestive heart failure, bacterial contamination, viral infections, and iron overload [39]. In this review, by reference to several clinical trials, we evaluated the incidence of blood transfusions in patients receiving ESAs and patients not receiving ESAs. Out of 17 randomized controlled trials included in this review, 14 trials compared the proportion of participants who received blood transfusions in the ESA group with the control group. Among the 14 trials, 13 trials showed that the number of participants who received at least one blood transfusion was lesser in the ESA group compared with the control group. In one trial, Cabanillas et al. showed no significant difference in the number of PRBC transfusion events per week between the two treatment groups (P =.089) [15]. However, the total number of blood transfusions administered per week was lesser in the ESA group compared with the control. The results of the trials are presented in Table 2.

**Table 2:** Proportion of participants who received a blood transfusion in ESA group vs Control group ESA = Erythropoiesis stimulating agent; PRBC = Packed red blood cell

Study	ESA	Proportion of participants who received transfusion in ESA group	Proportion of participants who received transfusion in Control group	P value
Aapro - 2008 [13]	Epoetin beta	14%	27%	<0.001
Blohmer - 2011 [14]	Epoetin alfa	10.7%	29.6%	<0.001
Cabanillas - 2012 [15]	Epoetin alfa	No difference	No difference	Number of PRBC units transfused per week 10.63 ± 6.29 vs. 13.11 ± 5.18; p <0.035
Engert - 2010 [16]	Epoetin alfa	63.3%	72.6%	<0.001
Fujisaka - 2011 [17]	Epoetin beta	4.5%	19.6%	=0.002
Gascon - 2019 [18]	Darbepoeitin alfa	23.4%	29.1%	=0.01
Gupta - 2009 [19]	Epoetin beta	15.5%	43.8%	<0.01
Hernandez - 2009 [20]	Darbepoeitin alfa	24%	41%	<0.001
Leyland- Jones - 2016 [22]	Epoetin alfa	5.8%	11.4%	<0.001
Pirker - 2008 [23]	Darbepoeitin alfa	17%	39%	<0.001
Platzbecker - 2017 [24]	Darbepoeitin alfa	36.1%	59.2%	=0.008
Pronzato - 2010 [25]	Epoetin alfa	3%	10.1%	=0.048
Ray-Coquard - 2009 [26]	Epoetin alfa	36.1%	58.1%	=0.001
Tsuboi - 2009 [28]	Epoetin beta	16.4% (after four weeks)	32.1% (after 4 weeks)	=0.046

The studies showed that ESAs are valuable drugs in treating anemia in cancer as they improve clinical outcomes significantly by reducing the need for blood transfusions in cancer patients.

Effectiveness of ESA Therapy: Mean Change in Hemoglobin Levels

A rise in hemoglobin levels in anemic cancer patients can improve physical and emotional well-being. An increase in energy levels, decreased fatigue, improved therapeutic outcomes, and reduced need for blood transfusions with an increase in hemoglobin levels lead to improved quality of life. The change in mean hemoglobin level from the baseline was compared between the ESA group and the control group in 10 trials. Out of 10 trials, eight trials showed a greater mean hemoglobin level in the ESA group than in the control group. In one trial, Cabanillas et al. showed no difference between the groups [15]. In another trial, Pirker et al. showed decreased mean hemoglobin levels in both groups [23]. However, the drop in hemoglobin level was less in the ESA group compared with the control group. The results of the trials are presented in Table 3.

**Table 3:** Mean change in Hb level in ESA group vs Control group Hb = Hemoglobin; ESA = Erythropoiesis stimulating agent

Study	ESA	Mean change in Hb level in ESA group	Mean change in Hb level in Control group	P value
Aapro - 2008 [13]	Epoetin beta	1.5 g/dl	-0.1 g/dl	<0.05
Cabanillas - 2012 [15]	Epoetin alfa	No difference	No difference	<0.21
Fujisaka - 2011 [17]	Epoetin beta	1.9 g/dl	0.0 g/dl	<0.001
Gascon - 2019 [18]	Darbepoeitin alfa	0.42 g/dl	-0.12 g/dl	<0.05
Gupta - 2009 [19]	Epoetin beta	1.55 g/dl	-1.50 g/dl	<0.01
Leyland- Jones - 2016 [22]	Epoetin alfa	11.6 (10.4) g/dl	10.9 (10.5) g/dl	<0.05
Pirker - 2008 [23]	Darbepoeitin alfa	-1.13 g/dl	-1.98 g/dl	<0.001
Pronzato - 2010 [25]	Epoetin alfa	10.6 g/dl to 12.3 g/dl	10.8 g/dl to 11.2 g/dl	<0.001
Smith Jr - 2008 [27]	Darbepoeitin alfa	0.73 g/dl	0.29 g/dl	<0.0001
Tsuboi - 2009 [28]	Epoetin beta	1.4 ± 1.9 g/dl	−0.8 ± 1.5 g/dl	< 0.001

Five trials also compared the proportion of patients with an increase in hemoglobin level between the ESA and the control group. All five trials showed that the proportion of patients with an increase in hemoglobin level was higher in the ESA group compared with the control group. The results are presented in Table 4.

**Table 4:** Percentage of patients with an increase in mean Hb level in ESA group vs Control group Hb = Hemoglobin; ESA = Erythropoiesis stimulating agent

Study	ESA	Percentage of patients with an increase in mean Hb level in ESA group	Percentage of patients with an increase in mean Hb level in the control group	P value
Aapro - 2008 [13]	Epoetin beta	68%	14%	<0.001
Blohmer - 2011 [14]	Epoetin alfa	51%	14%	<0.001
Platzbecker - 2017 [24]	Darbepoetin alfa	14.7%	0%	=0.016
Pronzato - 2010 [25]	Epoeitin alfa	62%	28%,	<0.01
Tsuboi - 2009 [28]	Epoetin beta	42.6%	1.8%	<0.001

The studies showed ESAs are effective in raising mean hemoglobin levels in anemic cancer patients.

ESA Therapy: Cardiovascular Adverse Effects 

ESA therapy is generally well tolerated. However, as with any other drug therapy, ESAs are associated with side effects. Cardiovascular side effects are a major concern with ESA therapy. In this present review, by reference to several clinical trials, we outlined the cardiovascular risks of ESAs in the treatment of cancer-related anemia. We involved 17 randomized controlled trials, including 5797 cancer patients with ESA and 4930 patients with controls. Cardiovascular side effects were observed in 16 trials in both the ESA group and the control group. In only one trial reported by Gupta et al., there were no cardiovascular side effects in either group [19]. The side effects observed were more in the ESA group than the control group in 14 trials. In two trials, Blohmer et al. [14] and Platzbecker et al. [24], cardiovascular side effects were comparable between two groups. Six-hundred seventy-seven patients out of 5797 patients in the ESA group experienced cardiovascular side effects compared to 418 patients out of 4930 patients in the control group. The type of side effects included venous thromboembolism, thrombophlebitis, hypertension, ischemic heart disease, cardiac failure, arrhythmia, arterial thromboembolism, and cardiac arrest. Venous thromboembolism is the most significant cardiovascular adverse effect attributed to ESA therapy as all the 16 trials had at least one event. A total of 286 patients experienced venous thromboembolism with ESAs compared to 170 patients with control. In all the 16 trials with cardiovascular side effects, venous thromboembolism events were greater in the ESA group compared to the control group. Overall survival between the ESA and control groups was compared in the 16 trials with cardiovascular side effects. Fifteen trials showed no significant difference in overall survival between the two groups. Only one trial, reported by Smith Jr et al., showed statistically significantly poorer survival in patients treated with darbepoetin versus placebo (P = .022) [27]. The cardiovascular side effects in ESA and control groups are presented in Table 5. 

**Table 5:** Cardiovascular side effects in ESA group vs Control group ESA = Erythropoiesis stimulating agent

Study	Participants with cardiovascular side effects in the ESA group	Total participants in the ESA group	Participants with cardiovascular side effects in the control group	Total participants in the control group	Type of cardiovascular side effects
Aapro - 2008 [13]	29	231	13	231	Pulmonary embolism, Thrombophlebitis, Deep venous thrombosis
Blohmer - 2011 [14]	2	128	3	129	Pulmonary embolism, Deep venous thrombosis
Cabanillas - 2012 [15]	5	55	2	54	Thrombotic events
Engert - 2010 [16]	6	648	5	655	Vascular disorders
Fujisaka - 2011 [17]	6	89	3	92	Hypertension, Pulmonary embolism
Gascon - 2019 [18]	261	1680	115	836	Thromboembolism, Hypertension, Ischemic heart disease, Cardiac failure
Gupta - 2009 [19]	0	58	0	57	NA
Hernandez - 2009 [20]	42	194	32	192	Arrhythmia, Cardiac failure, Hypertension, Ischemic heart disease, Thromboembolism, Cardiac arrest, Vascular disorders
Hoskin - 2009 [21]	8	151	7	149	Hypertension, Chest pain, Coronary artery disorder, Cardiac arrest, Thromboembolism
Leyland -Jones - 2016 [22]	29	1050	15	1048	Arterial thrombosis/embolism, Venous thrombosis/embolism, Acute coronary syndrome, Pulmonary embolism
Pirker - 2008 [23]	140	301	101	296	Thromboembolism, Arrhythmia, Heart failure, Coronary artery disorder, Thrombosis (arterial and venous), Hypertension
Platzbecker - 2017 [24]	7	98	8	48	Hypertension, Heart failure, Ischemic heart disease, Thromboembolism (venous and arterial)
Pronzato - 2010 [25]	8	107	7	109	Thrombovascular event
Ray-Coquard - 2009 [26]	5	110	4	108	Thrombovascular event
Smith Jr - 2008 [27]	106	517	87	472	Thromboembolic event, Arterial and venous thrombosis/embolism, Arrhythmia, Heart failure, Coronary artery disorders, Hypertension
Tsuboi - 2009 [28]	5	62	4	58	Thrombovascular event, Hypertension
Untch - 2011 [29]	18	318	12	396	Thromboembolism
Total	677	5797	418	4930	-

The studies showed ESA therapy is associated with cardiovascular side effects, including venous thromboembolism, thrombophlebitis, hypertension, ischemic heart disease, cardiac failure, arrhythmia, arterial thromboembolism, and cardiac arrest. An increase in these side effects was noted with aggressive ESA dosing to obtain higher hemoglobin levels, and in patients with preexisting uncontrolled hypertension. Venous thromboembolism is the most significant cardiovascular side effect attributed to ESA therapy, as it was found consistently in all the trials (16/16). The risk of thrombosis was higher in patients with preexisting ischemic heart disease and congestive heart failure and lower in young patients and patients with previous thromboembolic events who received anticoagulation prophylaxis. However, these side effects did not significantly impact overall survival as 15 out of 16 trials showed no change in overall survival between the ESA group and the control group.

Limitations

Our review has the following limitations. As this is a review at the study level, patient-level confounding factors could not be appropriately assessed and incorporated into the analysis. Some of the studies did not specify criteria for administering blood transfusions, which could impact ESA dosage and therapy. The association between the risk for cardiovascular adverse effects and the duration of ESA therapy could not be determined with the available data. The study did not distinguish incidental findings of cardiovascular adverse events, contributing to a bias of the reported incidence rates. Since we have taken the lower limit date as January 2008, we have not included the RCTs published before January 2008 that might be relevant to this analysis.

## Conclusions

Erythropoiesis stimulating agents (ESAs) are effective and valuable drugs in the treatment of anemia in cancer patients. ESAs improve clinical outcomes significantly by reducing the need for blood transfusions in cancer patients. ESAs are effective in increasing mean hemoglobin levels. However, ESA therapy is associated with cardiovascular adverse effects, including venous thromboembolism, thrombophlebitis, hypertension, ischemic heart disease, cardiac failure, arrhythmia, arterial thromboembolism, and cardiac arrest. These side effects are higher in patients with preexisting uncontrolled hypertension and with aggressive ESA dosing to achieve higher hemoglobin levels. Venous thromboembolism is the most significant cardiovascular adverse effect associated with the use of ESAs, but ESA therapy does not have a discernible role in overall survival. The risk-benefit ratio of ESA treatment in cancer-related anemia should be assessed carefully for each cancer patient. Caution is advised when using epoetin or darbepoetin in combination with thrombogenic chemotherapeutic agents or for cancer patients at high risk for thromboembolic events. The cellular and molecular processes and pathways by which ESAs affect thrombogenesis are not distinct. More research is needed on this subject. Elucidation of the role of ESAs in thrombosis will improve our ability to use ESAs rationally in the clinical setting and helps in the prevention and management of thromboembolism. Concerning potential risks and benefits, ESA therapy can be carried out in anemic cancer patients with careful assessment of thromboembolism risk factors and monitoring of hemoglobin levels.
